# Kainic acid-induced microglial activation is attenuated in aged interleukin-18 deficient mice

**DOI:** 10.1186/1742-2094-7-26

**Published:** 2010-04-14

**Authors:** Xing-Mei Zhang, Tao Jin, Hernan Concha Quezada, Eilhard Mix, Bengt Winblad, Jie Zhu

**Affiliations:** 1 Department of Neurobiology, Care Sciences and Society, Karolinska Institute, Stockholm, Sweden; 2 Department of Neurology, the First Hospital of Jilin University, Changchun, China; 3 Center for Infectious Medicine, Department of Medicine, Karolinska Institute, Stockholm, Sweden; 4 Department of Neurology, University of Rostock, Rostock, Germany

## Abstract

**Background:**

Previously, we found that interleukin (IL)-18 deficiency aggravates kainic acid (KA)-induced hippocampal neurodegeneration in young C57BL/6 mice due to an over-compensation by IL-12. Additionally, IL-18 participates in fundamental inflammatory processes that increase during aging. In the present study, we were interested in the role of IL-18 in KA-induced neurodegeneration in aged female C57BL/6 mice.

**Methods:**

Fifteen aged female IL-18 knockout (KO) and 15 age-matched wild-type (WT) mice (18 to 19 months old) were treated with KA at a dose of 25 mg/kg body weight intranasally. Seizure activities and behavioral changes were rated using a 6-point scoring system and open-field test, respectively. Seven days after KA treatment, degenerating neurons were detected by Nissl's method and Fluoro-Jade B staining; and microglial activation was analyzed by immunohistochemistry and flow cytometry.

**Results:**

Aged female IL-18 KO and WT mice showed similar responses to treatment with KA as demonstrated by comparable seizure activities, behavioral changes and neuronal cell death. However, aged female IL-18 KO mice failed to exhibit the strong microglial activation shown in WT mice. Interestingly, even though the number of activated microglia was less in KA-treated IL-18 KO mice than in KA-treated WT mice, the proportion of microglia that expressed the cytokines tumor necrosis factor (TNF)-α, IL-6 and IL-10 was higher in KA-treated IL-18 KO mice.

**Conclusion:**

Deficiency of IL-18 attenuates microglial activation after KA-induced excitotoxicity in aged brain, while the net effects of IL-18 deficiency are balanced by the enhancement of other cytokines, such as TNF-α, IL-6 and IL-10.

## Background

Administration of kainic acid (KA), an analog of the excitotoxin glutamate, to rodents results in neuronal death and seizures [1], which provides a well-characterized model for studies of human neurodegenerative diseases [2-4]. Synthesis and release of cytokines and other inflammatory factors by glial cells influence the survival and repair of hippocampal neurons after injury [5,6].

Interleukin (IL)-18, first isolated as interferon-γ inducing factor [7], is related to the IL-1 family by mechanism of origin, receptor structure, and signal transduction pathways utilized [8]. IL-18 serves as a link between innate and adaptive immune responses, through mechanisms such as stimulating the expression of adhesion molecules, inducing the production of chemokines (IL-8) and cytokines (tumor necrosis factor and IL-6), stimulating the activity of NK cells, stimulating the expression of Fas ligand on T and NK cells and the cytotoxic activity of CD8^+ ^effector T cells, stimulating Th1 responses in combination with IL-12, stimulating Th2 responses in combination with IL-2, and stimulating Th17 responses in combination with IL-23 [9]. IL-18 and IL-18 receptor (IL-18R) mRNA have been detected in brain tissue from adult rats and in cultured astrocytes and microglia [10,11]. However, the effects of IL-18 in neurodegeneration are complicated. Our previous study showed that young (six to eight weeks old) IL-18 knockout (KO) mice were more sensitive to KA administration than wild-type (WT) animals with normal expression of IL-18, although exogenous IL-18 administration could aggravate KA-induced neurodegeneration [12].

We also found that aged female mice were more susceptible to KA-induced excitotoxicity than male mice, which resembles the situation for humans [13]. It has also been suggested that IL-18 participates in fundamental inflammatory processes that increase during aging [14]. In the present study, we therefore aimed to investigate the role of IL-18 in KA-induced neurodegeneration in aged female C57BL/6 mice. Our results demonstrate that deficiency of IL-18 attenuates microglial activation after KA-induced excitotoxicity in aged brain.

## Methods

### Animals

Fifteen aged female IL-18 KO and 15 age-matched C57BL/6 WT mice (18 to 19 months old) were used in the present study. The mice were purchased from the Jackson Laboratory (Bar Harbor, ME, USA) at age of 2 months and kept at the animal facilities of Karolinska University Hospital, Huddinge, Sweden until the designed ages for experiments. All mice were housed on a 12 h light-dark schedule with water and food available ad libitum. The KA-induced excitotoxic model in mice was approved by the South Stockholm Research Animal Ethics Committee, Huddinge County Court, Stockholm, Sweden. All efforts were made to minimize the number of animals used and their sufferings.

### KA administration and assessment of seizure activity

Mice were partially anesthetized with Isoflurane (Abbott Laboratories, Kent, UK) and held on their backs by hand. KA, (Opika-1, Ocean Produce International, Shelburne, Nova Scotia, Canada) dissolved in saline, (10 mg/1.3 ml) was slowly and gently dropped by micropipette into the nostrils of the mice at a dose of 25 mg/kg body weight as described previously [15]. Four IL-18 KO mice and 5 WT mice were administrated with physiological saline as control. Mice were observed by two different examiners continuously for 5 hours to record the onset and extent of seizure activity. Seizures were rated according to the criteria described by Ben-Ari [16] with modifications: 0, normal; 1, immobilization; 2, rearing and falling; 3, seizure for less than 1 h; 4, seizure for 1-3 h; 5, seizure for more than 3 h; and 6, death.

### Open-field test with zone monitoring

Open-field activity was measured one day before and six days after KA administration in 8 IL-18 KO mice and 7 WT mice. The open field test was used to measure exploratory behaviour and spontaneous motor activity of the animals. The apparatus consisted of four identical Plexiglas boxes (34 × 34 × 18 cm) with a lower and a higher row of infrared sensitive photocells connected to a computer that registered the interruptions of these cells which corresponded to the horizontal and vertical activities of the animals inside of the arena. Light bulbs (25 W) provided the illumination for each arena. Four mice were tested simultaneously, one per arena. At the beginning each mouse was placed into the center of open-field arena and its movement was recorded every 5 min during 60 min. The overall locomotion and the number of times the animal lifted its fore paws and stood on its back feet (rearing) were analyzed. All tests were carried out between the hours of 9:00 and 15:00.

### Histopathological analysis

All mice were anesthetized with sodium pentobarbital and transcardially perfused with phosphate-buffered saline (PBS) seven days after the administration of KA. The left hemisphere of each brain was fixed in 4% paraformaldehyde and kept in 10% sucrose until being frozen, and the right hippocampus was dissociated and put into Krebs-Ringer buffer (KRB) solution [120 mM NaCl, 5 mM KCl, 1.2 mM KH_2_PO_4_, 25 mM NaHCO_3_, 14 mM D-glucose, 2.5 mM MgSO_4_, 0.3% bovine serum albumin (BSA)] for isolation and flow cytometric analysis of microglia. Coronal sections (12-μm slices) from -1.15, - 1.94 and - 2.80 mm, respectively, relative to the bregma were prepared according to the information in Franklin's brain atlas [17]. Sections were stained by Nissl's method and Fluoro-Jade B (FJB) (Histo-Chemo, Inc., Jefferson, AR) staining to evaluate degenerating neurons. FJB is an anionic fluorescein derivative useful for the histological staining of neurons undergoing degeneration. For assessment of severity and extent of neurodegeneration in the hippocampus after Nissl's staining, sections were scored using a semiquantitative grading system: 0, normal; 1, slight shrinkage of neurons (1 - 4% pyknotic neurons in area CA3); 2, moderate shrinkage of neurons (5 - 15% pyknotic neurons in area CA3); 3, severe shrinkage of neurons (more than 15% pyknotic neurons in area CA3); 4, slight loss of neurons (5 - 10% neuron loss in area CA3); 5, moderate loss of neurons (11 - 40% neuron loss in area CA3); and 6, severe loss of neurons (more than 40% neuron loss in area CA3). Using a blinded protocol, two different examiners duplicated the counting.

### Immunohistochemistry of brain sections

Frozen hippocampal sections were prepared as described for histopathological analysis. After washes with Tris buffer, the sections were blocked by "protein block" (DAKO, Glostrup, Denmark) at room temperature for 30 min. Subsequently, they were exposed to rat antibodies to CD11b (1:100, Serotec, Oxford, UK) and rabbit antibodies to glial fibrillary acidic protein (GFAP) (1:2000; DAKO), respectively, followed by staining with the avidin-biotin technique (Vectastain Elite Kit, Vector Labs, Burlingame, CA, USA). Peroxidase-substrate solution DAB (Sigma-Aldrich, Stockholm, Sweden) was added until the desired intensity of yellow color developed. Omission of primary antibodies served as negative control. Slides were observed using a Nikon Eclipse E800-U microscope (Nikon, Japan). Positive immunostaining of cells was rated using a semiquantitative scale - baseline staining (·), mild response (+), moderate response (+ +), intense response (+ + +) - by two observers, independently. Criteria for each scale followed the description by Colburn et al [18].

### Isolation and flow cytometric analysis of microglia

As mentioned above, seven days after KA treatment, the right hippocampus of each brain was collected and put into KRB solution for isolation and flow cytometric analysis of microglia. The hippocampi were dissected and dissociated by pipetting. Next, after trypsinization at 37°C for 15 min, fetal bovine serum (10%, final concentration) was added to inactivate trypsin activity. The tissues were then dissociated with repeated pipetting in KRB solution containing DNase I (Sigma-Aldrich). Cell suspensions were passed through a 70-μm pore-size strainer and spun down. The cell pellets were resuspended in 30% Percoll in PBS and centrifuged at 500 × g for 20 min. The resulting pellets were resuspended, passed through a 40-μm pore-size strainer, collected, and stained for flow cytometry.

Microglia-enriched cell suspensions were washed with PBS containing 1% BSA (BSA/PBS), permeabilized, fixed and incubated with the following antibodies: APC-labeled rat anti-mouse CD11b (Caltag, Burlingame, CA, USA), either PerCP-labeled rat anti-mouse CD45 (Serotec), RPE-labeled mouse anti-mouse major histocompatibility complex (MHC)-II I-Ak (Serotec) and FITC-labeled rat anti-mouse tumor necrosis factor-α (TNF-α) (Becton Dickinson Biosciences, San José, CA, USA), or RPE-labeled rat anti-mouse IL-6 (BD Pharmingen, San Diego, CA, USA) and FITC-labeled rat anti-mouse IL-10 (Pharmingen). FITC-, RPE-, PerCP (Serotec), and APC-(Caltag) labeled rat IgG were used as isotype controls to set the gates. Cell size, granularity, and fluorescence intensity were measured using FACScalibur flow cytometer (Becton Dickinson). A minimum of 5 × 10^3 ^cells were analyzed with CellQuest software (Becton Dickinson). Microglia from all four groups (WT mice with or without KA treatment, and IL-18 KO mice with or without KA treatment) were collected and analyzed on the same day with the same cytometer settings.

### Data presentation and statistics

The Mann-Whitney U test was used to analyze the clinical, histopathological and immunohistochemical data, and one-factor analysis of variance (ANOVA) was used to analyze flow cytometric and behavioral results. Data are presented as mean ± SD. P < 0.05 was considered as significant in all tests. Graphics and calculations were performed using GraphPad PRISM version 5.0 (GraphPad, USA), and SPSS Version 15.0 (SPSS Inc, USA).

## Results

### IL-18 deficiency does not affect KA-induced seizure activity in aged female mice

Intranasal application of KA (25 mg/kg bodyweight) induced various clinical symptoms in both IL-18 KO and WT female mice within 30 min, from behavioral arrest, facial myoclonus, rearing and falling to severe clonic convulsions. Three mice of each group died of serious seizures including status epilecticus (Table 1). The severity of seizures did not differ significantly between the two groups. The medians of clinical scores for the IL-18 KO and WT mice were 3 (1-5) and 5 (1-6), respectively. Neither IL-18 KO nor WT mice given saline intranasally showed any clinical signs.

**Table 1:** Survey of seizure activity in mice after KA treatment.

	Groups	
	
Seizure Activity Variables	KO (n = 11)	WT (n = 10)
Tonic/clonic twitching without seizures	4/11	2/10
Seizures < 1 h	2/11	1/10
Seizures 1-3 h	1/11	2/10
Seizures > 3 h	1/11	2/10
Death due to seizures	3/11	3/10

Clinical scores (Medians with percentiles)	3 (1-6)	5 (1-6)

Female IL-18 KO and WT mice, 18 to 19 months of age, were treated with KA at a dose of 25 mg/kg bodyweight intranasally. Mice were monitored continuously for 5 h to register the onset and extent of seizure activity. KA treatment resulted in various clinical symptoms in both IL-18 KO and WT mice. However, the severity of seizure did not differ significantly between the two groups.

### IL-18 deficiency does not influence KA-induced behavioral changes in aged female mice

**Figure 1:**
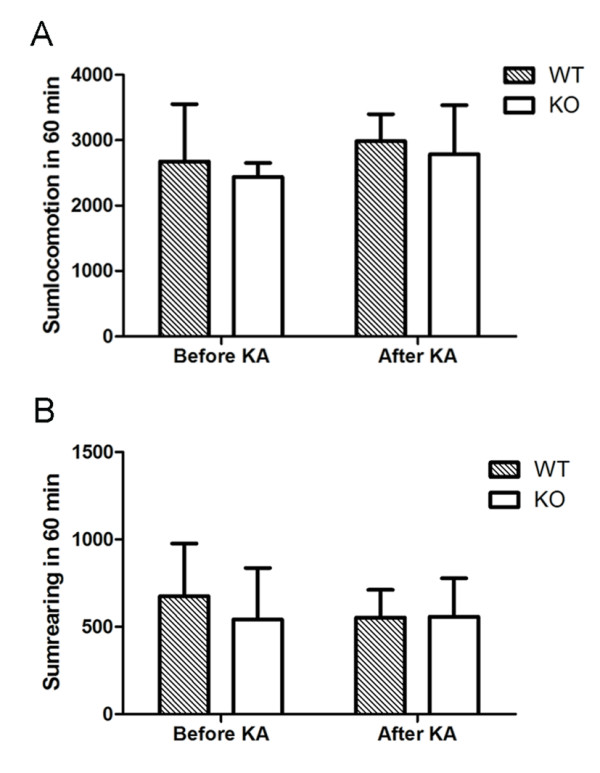
**Comparisons of locomotion (A) and rearing (B) activity 1 day before and 6 days after KA administration in IL-18 KO (n = 8) and WT (n = 7) aged (18 to 19 months old) female mice**. IL-18 deficiency does not influence KA-induced behavioral changes in aged female mice.

Six days after KA treatment, IL-18 KO and WT mice showed a similar habituation profile during a 60-min open-field test period. KA treatment did not affect locomotion or rearing either in WT or KO mice. Moreover, there was no significant difference between WT and IL-18 KO mice either before or after KA delivery, indicating that IL-18 deficiency does not affect KA-induced spontaneous activity and exploratory behaviour in aged female mice (Fig. 1A and 1B).

### Aged female IL-18 KO mice and WT mice show similar KA-induced hippocampal neurodegeneration

**Figure 2:**
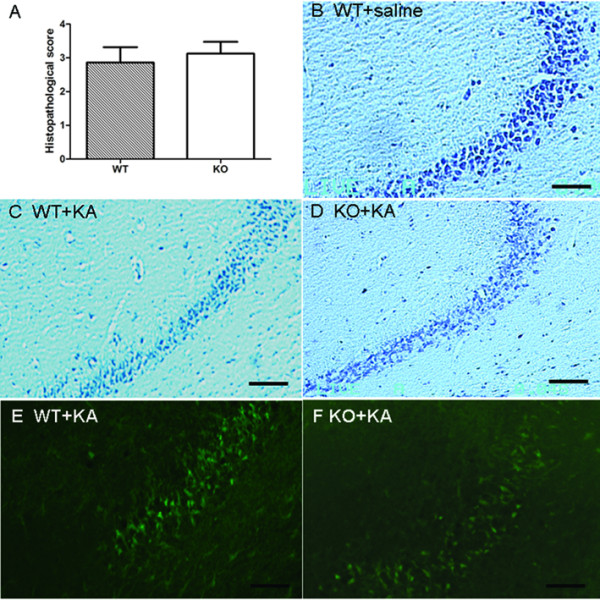
**Histochemical staining of coronal mouse hippocampal sections (showing CA3 area) of aged female mice after KA treatment**. Sections were prepared 7 days after administration of KA and stained by Nissl's method or with Fluoro-Jade B (FJB) staining. The sections were scored using a semi-quantitative grading system as described in the Methods. Histopathological scores are presented in the surviving IL-18 KO mice (n = 8) and WT mice (n = 7) (A). There was no difference in pathological changes after KA treatment between IL-18 KO and WT mice. Representative sections of both groups of mice are presented (B, saline-treated control WT mice; C, Nissl-stained KA-treated WT mice; D, Nissl-stained KA-treated IL-18 KO mice; E, FJB-stained KA-treated WT mice; F, FJB-stained KA-treated IL-18 KO mice). Bars = 100 μm.

Seven days after KA administration, selective hippocampal neurodegeneration was observed. Most brain regions showed no neuropathological changes; the exceptions being the CA3 area of hippocampus, which was consistent with our previous studies [15]. The average histopathological scores at the center (-1.94 mm to bregma) of the hippocampus are presented in Fig. 2A. Continuous sectioning showed that samples at three levels (anterior, -1.15 mm to bregma, middle, -1.94 mm and posterior, -2.80 mm) demonstrated the same overall changes in the hippocampus (data not shown). Seven days after KA treatment, at all three hippocampal levels, IL-18 KO mice (Fig. 2D) and WT mice (Fig. 2C) exhibited similar degrees of neurodegeneration. A control group treated with saline showed no pathological changes in the hippocampus (Fig. 2B). The extent of KA-induced hippocampal neuronal degeneration in IL-18 KO mice and WT mice was further confirmed using FJB staining (Figs. 2E and 2F). In both IL-18 KO mice and WT mice, FJB-positive neurons were limited to the CA3 region of the hippocampus, with no difference between the two groups of mice.

### KA-induced hippocampal astrogliosis is not changed by IL-18 deficiency in aged female mice

**Figure 3:**
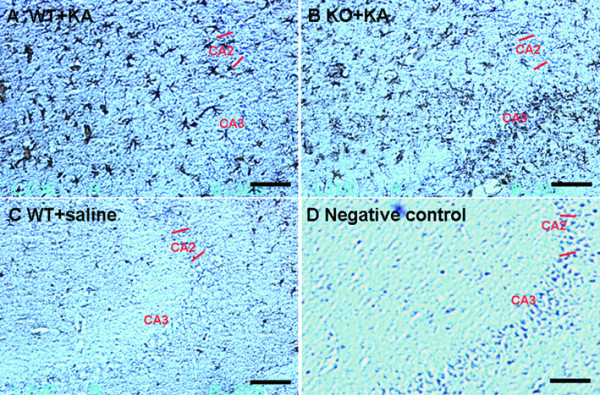
**GFAP expression in hippocampi of aged female mice detected by immunohistochemistry**. GFAP positive cells were shown as dark brown in color. Seven days after KA treatment, the intensity of GFAP staining in IL-18 KO mice (B) was similar to that in WT mice (A) with similar neuropathological changes. Very few reactive astrocytes were found in the hippocampi of saline-treated mice (C) and negative controls, with omission of the primary antibody, showed no positive staining (D). Bars = 100 μm.

Excitotoxicity causes astrogliosis in brain, which is thus a feature of KA-induced neurodegeneration in hippocampus. We evaluated astrogliosis by detection of the expression of GFAP, a marker for astrocytes. Seven days after KA treatments, GFAP immunoreactivity was similarly enhanced (mild to moderate response) in the hippocampi of IL-18 KO (Fig. 3B) and WT mice (Fig. 3A). Reactive astrocytes were found throughout the hippocampus, including CA1, CA2, CA3 and the dentate gyrus, and were particularly prominent in the CA3 area. Very few reactive astrocytes were found in the hippocampi of saline-treated mice (Fig. 3C, baseline staining), and negative controls with omission of the primary antibody showed no positive staining (Fig. 3D). Positive immunostaining of cells was rated using the semiquantitative scale mentioned in Methods. There was no difference in KA-induced astrogliosis between the two groups of mice (data not shown).

### IL-18 deficiency attenuates KA-induced microglial activation in aged female mice

**Figure 4:**
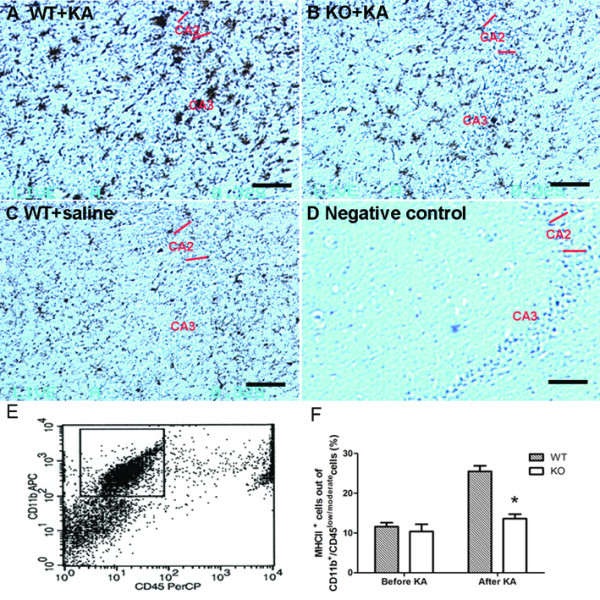
**CD11b and MHC-II expression in hippocampi of aged female mice detected by immunohistochemistry and flow cytometry**. Seven days after KA treatment, the number of activated microglia was greater in WT mice (A) than in IL-18 KO mice (B) with similar neuropathological changes. Very few activated microglia were found in the hippocampi of saline-treated mice (C) and negative controls with omission of the primary antibody showed no positive staining (D). CD11b positive cells were shown as dark brown in color. By flow cytometry, microglia of hippocampi were identified as a homogeneous population of CD11b^+^/CD45^low/moderate^cells (E). The proportion of MHC-II positive cells was higher in WT mice (n = 5) than in IL-18 KO mice (n = 6) after KA treatment. Before KA delivery, there was no difference of MHC-II positive cells between the two groups (F). *P < 0.05. Bars = 100 μm.

Microglia are the main effector cells of the immune system in the central nervous system (CNS). We evaluated microglial activation by detection of the expression of CD11b and MHC-II by immunohistochemistry and flow cytometry. CD11b can detect both resting and activated microglia, while strong CD11b-immunreactive microglia show a marked cellular hypertrophy with thicker and shorter processes. Seven days after KA delivery, although both groups of mice exhibited microglial activation, the number of activated microglia of WT mice (Fig. 4A) was consistently higher than in IL-18 KO mice (Fig. 4B). Very few activated microglia were found in the hippocampi of saline-treated mice (Fig. 4C) and the negative controls with omission of the primary antibody showed no positive staining (Fig. 4D).

By flow cytometry, microglia of hippocampi were identified as a homogeneous population of CD11b positive cells with low/moderate CD45 staining (CD11b+/CD45low/moderate cells, Fig. 4E). The possible macrophage contamination was excluded by deletion of the high CD45 staining cells. Similar to the findings of immunohistochemistry the number of activated microglia (MHC-II positive cells) in WT mice was significantly higher than in the IL-18 KO mice 7 days after KA treatment (Fig. 4F). Before KA treatment, there was no difference in the numbers of MHC-II positive cells between the two groups.

### Expression of TNF-α, IL-6 and IL-10 is increased in IL-18 KO mice after KA treatment

**Figure 5:**
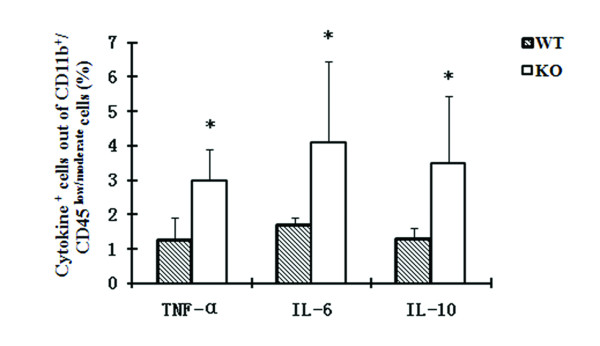
**TNF-α, IL-6 and IL-10 expression in activated microglia of aged female mice detected by flow cytometry**. Seven days after KA treatment, the percentages of TNF-α, IL-6 and IL-10 positive cells among CD11b^+^/CD45^low/moderate ^gated cells increased significantly in IL-18 KO mice (n = 6) compared to WT mice (n = 5). Mean percentages of cytokine-positive cells and SD values are presented. *P < 0.05.

We assessed microglial expression of the cytokines TNF-α, IL-6 and IL-10 using flow cytometry. The results of flow cytometric analyses of cytokine expression are shown as the percentage of cytokine-positive cells in the CD11b+/CD45low/moderate population. Seven days after KA treatment, the expression of TNF-α, IL-6 and IL-10 increased significantly in IL-18 KO mice compared with WT mice (Fig. 5). Before KA treatment, there was no difference in TNF-α, IL-6 or IL-10 expression between IL-18 KO and WT mice (data not shown).

## Discussion

In the present study, we investigated the role of IL-18 in the pathogenesis of KA-induced excitotoxic neurodegeneration in the aged situation by using IL-18-deficient aged (18 to 19 months old) mice and age-matched WT mice. The study was motivated by the fact that most common neurodegenerative disorders such as Alzheimer's disease and Parkinson's disease are typically diseases of higher ages and by the hypothesis that findings of KA-induced neurodegeneration of young individuals may deviate from findings in older subjects. Since previously we also found that aged female C57BL/6 mice were more sensitive to KA treatment than aged male mice [13], we performed this study on female animals.

Our results show that IL-18 deficiency does not influence susceptibility to KA-induced injury in aged female mice, since KA-induced seizures, behavioral changes and histopathological changes including astrogliosis were similar in IL-18 KO mice and WT controls. However, we found also that aged IL-18-deficient mice show lower microglial activation in response to KA than do WT mice as demonstrated by lower numbers of CD11b-positive and MHC-II-positive cells. On the other hand, in these activated microglia the percentages of TNF-α-, IL-6- and IL-10-positive cells were significantly higher in IL-18 KO mice than in WT mice. These results are in contrast to our previous findings in KA-induced excitotoxic neurodegeneration of IL-18-deficient young mice [12]. Young (6 to 8 weeks old) IL-18 KO mice were more sensitive to KA administration than age-matched animals with normal expression of IL-18. In that situation we concluded that excitotoxic injury in IL-18 deficient mice might be due to overcompensation by other microglia-derived, disease-promoting factors, of which IL-12 is one candidate. Our present finding that aged IL-18 KO mice display similar susceptibility to KA treatment as WT mice might be due to the balancing influence of other microglia-derived cytokines such as TNF-α, IL-6 and IL-10.

However, we also found in young animals that exogenous IL-18 administration could aggravate KA-induced neurodegeneration, when high doses of IL-18 are applied [12]. A recent study of Jeon and co-workers gave hints in the same direction by showing that levels of IL-18 and its receptor in hippocampal homogenates increased from day 1 post-KA onward [19]. Other researchers have found that lower IL-18 concentrations are associated with improved activities of daily living in 65- to 80-year-old men, suggesting that IL-18 might play an active role in age-related functional impairment [20]. Moreover, increased hippocampal concentrations of the proinflammatory cytokines IL-1α, IL-18 and IFN-γ are accompanied by deficits in long-term potentiation in older rats [21]. The imbalance between pro-inflammatory and anti-inflammatory cytokines in the aged brain significantly contributes to age-related deficits in synaptic function [22].

Upon neuronal injury, microglia, as the main effector cells of the immune system in the CNS, acquire changes in morphology and expression of surface antigens and soluble molecules [23]. In the present study, lower KA-induced microglial activation in the hippocampi of aged IL-18 KO mice was found compared with WT as detected by immunohistochemistry and flow cytometry. This finding is in agreement with studies of Sugama and co-workers, who found that stress-induced microglial activation is reduced in IL-18 KO mice [24]. IL-18 null mice also show diminished microglial activation and reduced dopaminergic neuron loss following acute 1-methyl-4-phenyl-1,2,3,6-tetrahydropyridine treatment [25]. Interestingly, even though the number of activated microglia is smaller in KA-treated IL-18 KO mice than in KA-treated WT mice, the proportion of microglia cells that express the cytokines TNF-α, IL-6 and IL-10 is higher in the KA-treated IL-18 KO mice, which may account for the similar neuropathological and clinical outcome of the IL-18-deficient, aged mice.

## Conclusion

This study demonstrates that deficiency of IL-18 attenuates microglial activation in KA-induced excitotoxicity in aged brain, while the net effects of IL-18 are balanced by other cytokines, such as TNF-α, IL-6 and IL-10. IL-18 may participate in KA-induced hippocampal neurodegeneration in young animals, but IL-18 does not seem to represent a key cytokine in this process in aged individuals.

## Competing interests

The authors declare that they have no competing interests.

## Authors' contributions

X-MZ performed the majority of experiments and data analysis, and wrote the initial version of the manuscript. TJ made substantial contributions to KA administration and microglia isolation. HCQ was involved in microglia/cytokines detection by flow cytometry. EM was involved in critical revisions of the manuscript. BW and JZ supervised all experimental procedures. All of the authors have read and approved the final version of the manuscript.
